# Mitochondrial Reactive Oxygen Species, Insulin Resistance, and Nrf2-Mediated Oxidative Stress Response—Toward an Actionable Strategy for Anti-Aging

**DOI:** 10.3390/biom13101544

**Published:** 2023-10-19

**Authors:** Shuya Kasai, Daichi Kokubu, Hiroki Mizukami, Ken Itoh

**Affiliations:** 1 Department of Stress Response Science, Center for Advanced Medical Research, Hirosaki University Graduate School of Medicine, 5 Zaifu-cho, Hirosaki 036-8562, Japan; kasai-s@hirosaki-u.ac.jp; 2 Department of Vegetable Life Science, Hirosaki University Graduate School of Medicine, 5 Zaifu-cho, Hirosaki 036-8562, Japan; daichi_kokubu@hirosaki-u.ac.jp; 3 Diet & Well-being Research Institute, KAGOME CO., LTD., 17 Nishitomiyama, Nasushiobara 329-2762, Japan; 4 Department of Pathology and Molecular Medicine, Hirosaki University Graduate School of Medicine, 5 Zaifu-cho, Hirosaki 036-8562, Japan; hirokim@hirosaki-u.ac.jp

**Keywords:** mitochondrial ROS, Nrf2, insulin resistance, diabetes, adipose tissue

## Abstract

Reactive oxygen species (ROS) are produced mainly by mitochondrial respiration and function as signaling molecules in the physiological range. However, ROS production is also associated with the pathogenesis of various diseases, including insulin resistance (IR) and type 2 diabetes (T2D). This review focuses on the etiology of IR and early events, especially mitochondrial ROS (mtROS) production in insulin-sensitive tissues. Importantly, IR and/or defective adipogenesis in the white adipose tissues (WAT) is thought to increase free fatty acid and ectopic lipid deposition to develop into systemic IR. Fatty acid and ceramide accumulation mediate coenzyme Q reduction and mtROS production in IR in the skeletal muscle, while coenzyme Q synthesis downregulation is also involved in mtROS production in the WAT. Obesity-related IR is associated with the downregulation of mitochondrial catabolism of branched-chain amino acids (BCAAs) in the WAT, and the accumulation of BCAA and its metabolites as biomarkers in the blood could reliably indicate future T2D. Transcription factor NF-E2-related factor 2 (Nrf2), which regulates antioxidant enzyme expression in response to oxidative stress, is downregulated in insulin-resistant tissues. However, Nrf2 inducers, such as sulforaphane, could restore Nrf2 and target gene expression and attenuate IR in multiple tissues, including the WAT.

## 1. Introduction

OxPhos proteins in the mitochondria are a mosaic of nuclear- and mitochondria-encoded proteins except for complex II, and electron transfer is not perfectly shielded. Therefore, a small percentage of electrons that evade from an electron transfer chain (ETC) cause a one-electron reduction in O_2_ either in the mitochondrial matrix or intermembrane space, leading to the generation of superoxide anion radicals. Superoxides are further reduced by superoxide dismutases SOD2 and SOD1 localized in the matrix or the intermembrane space, respectively, leading to the generation of hydrogen peroxides (H_2_O_2_) [1]. Mitochondrial H_2_O_2_ is reduced into water by glutathione peroxidase-1 and -4 (GPX-1/-4) and peroxiredoxin-3 (PRDX3) [2]. The generation of mitochondrial reactive oxygen species (mtROS) increases when the ETC is reduced, either by the increased influx of electrons to the ETC, such as in oxygen reperfusion injuries, or by the slowdown/inhibition of electron transfer, such as in decreased demand for ATP. These mtROS oxidize cellular components and cause damage when produced in excess, such as in ischemia-reperfusion injuries [3]. However, a small amount of mtROS acts as a signal transducer. A slight mismatch between mitochondria and nucleus-encoded proteins also leaks more electrons and, therefore, more mtROS [4]. The mtROS then induce compensatory mechanisms that increase the amount of mitochondrial DNA (mtDNA) needed to supply the mitochondrial ETC enzymes. Other pieces of evidence also support the concept that mtROS generated during exercise or other stimuli act as signal transducers for mitohormesis, which confers antioxidant defense capacities as an adaptive response [5]. Furthermore, mtROS are involved in multiple chronic diseases such as type 2 diabetes (T2D) as well as neurodegenerative diseases [6].

NF-E2-related factor 2 (Nrf2) is a transcription factor that regulates gene expression to elicit a defense system against oxidative stress in multicellular animals that evolved after metazoans [7,8]. Nrf2 activation is regulated by Kelch-like ECH-associated protein 1 (Keap1), which functions as a sensor for electrophiles and oxidative stress (molecular machinery is described in Section 4.1). Nrf2 target genes take part in detoxification, glutathione (GSH) synthesis, redox homeostasis, and proteostasis to counteract oxidative stress, mainly in the cytoplasm. Dietary Nrf2 inducers (e.g., sulforaphane)-triggered Nrf2-mediated oxidative stress response induction could modulate or prevent such disease progression. In fact, the Nrf2 inducer is used as a therapeutic agent for neurodegeneration and kidney disease associated with oxidative stress and inflammation [9,10,11]. On the other hand, somatic mutations that cause Nrf2 activation were found in tumors such as non-small cell lung epithelial and esophagus cancers, and Nrf2 plays tumorigenic functions [12,13,14]. The pro-survival function of Nrf2 is reportedly utilized in other cancers as well, and Nrf2 inhibitors are useful in such cases.

A hypothesis argues that transient insulin resistance (IR) evolved to protect against oxidative stress [15], but prolonged IR is a risk factor for developing chronic diseases such as type 2 diabetes and cardiovascular diseases [16]. In this review, after briefly summarizing the early events and molecular mechanisms of IR, including metabolic alteration of glucose, fatty acid, and branched-chain amino acids (BCAAs) in insulin-sensitive tissues, we focused on the role of mitochondrial dysfunction and mtROS production as well as the Nrf2-mediated adaptive response by dietary Nrf2 inducers and exercise in developing IR.

## 2. Mitochondrial Dysfunction at the Common Pathway in T2D Pathogenesis

### 2.1. T2D Etiology

T2D is often associated with obesity and old age, manifesting impaired glucose tolerance and increased fasting blood glucose levels. Obesity, IR, and hyperinsulinemia together precede T2D as a triad [16]. However, the members of this triad are interrelated, and determining the initial cause that ultimately leads to T2D is difficult. In fact, chronic hyperinsulinemia leads to IR [17,18], and genetic manipulation-induced IR causes hyperinsulinemia in mice. The concept that overflowed free fatty acids, as a result of overnutrition, would cause IR (usually called lipotoxicity) has been heavily debated [19]. However, the precise underlying mechanisms of IR are unclear. Nevertheless, IR is the first apparent event among the triad appearing in lean T2D, and mitochondrial dysfunction could precede IR in genetically predisposed or aged lean T2D (i.e., mitochondrial dysfunction -> aberrant fatty acid accumulation in the tissue -> IR) [20,21]. T2D develops against the background of family history, and many T2D-linked genetic loci were identified by a genome-wide association study (GWAS), providing hints to T2D etiology [22,23]. Hereafter, we will mainly discuss obese T2D and IR etiology.

### 2.2. IR Mechanisms as the Early Event of Obese T2D

**Figure 1:**
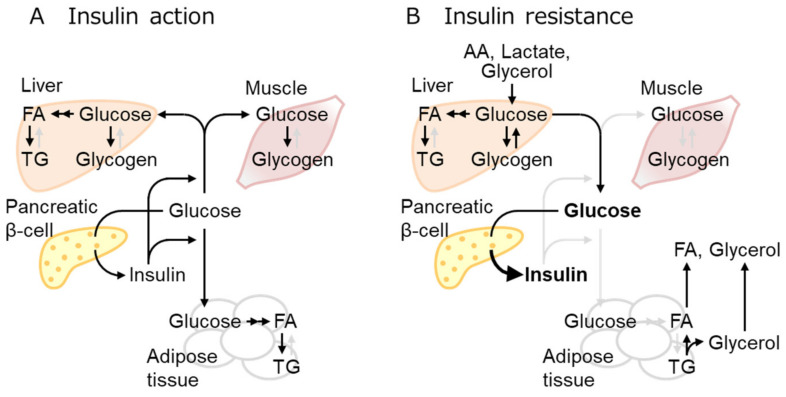
Insulin effect and resistance in multiple tissues. (**A**) Increased blood glucose stimulates insulin secretion, thereby increasing glucose uptake mainly in the muscles, adipose tissue, and liver, where glucose is stored as glycogen or converted to fatty acid (FA), then triglyceride (TG). (**B**) Insulin resistance denotes the loss of the insulin effect, glucose uptake reduction in insulin-sensitive tissues, and impaired suppression of hepatic gluconeogenesis, leading to hyperglycemia. Three-carbon substrates, including amino acids (AA), lactate, and glycerol, are used for gluconeogenesis. Overnutrition increases fat mass and circulating FA over the adipose tissue capacities. Active and inactive metabolic pathways were indicated with black and pale gray arrows, respectively.

Insulin exerts overlapping but different roles in each organ. IR designates the defect of insulin to take action, although it generally refers to the inability of a certain amount of insulin to lower blood glucose levels mainly by glucose uptake in the muscles, liver, and fat as well as to suppress glucose production in the liver [16] (Figure 1). The main effect of insulin in the liver comprises the suppression of glucose production (as well as the enhancement of glucose use or storage as glycogen) and the enhancement of *de novo* lipogenesis. Glucose-related lipogenesis enhancement and lipolysis suppression are the main functions of insulin in adipose tissue. Extensive crosstalk between insulin-sensitive tissues is a feature of energy metabolism. Insulin-induced gluconeogenesis suppression is reportedly caused by liver acetyl-CoA reduction due to the insulin-mediated inhibition of lipolysis in the adipose tissue [24]. Thus, failure of insulin-mediated gluconeogenesis suppression is actually caused by the defect of insulin-mediated lipolysis inhibition in the adipose tissue. However, insulin directly activates lipogenesis in the liver by activating AKT-mediated mTORC1 and FOXO1 inhibition [25]. In T2D, only the former insulin effect is often impaired, called selective IR [26,27]. Experiments in mice indicated that IR in one tissue is transmissible to other tissues, leading to systemic IR. For example, recent observations revealed that IR in the white adipose tissues (WAT) results in subsequent T2D development [16].

Obesity is often associated with adipose tissue-related inflammation. However, inflammation might not be the initial cause of IR, although it exacerbates IR, ultimately leading to T2D [16,28]. A growing body of evidence suggests that IR in T2D is not caused by the defect of proximal insulin signaling (such as the defect of AKT phosphorylation), but by the defect of distal insulin signaling located more closely to the final biological response (such as defects of GLUT4 trafficking to the plasma membrane) [16]. Insulin receptors and proximal signaling molecules (such as AKT) are available in excess, and the downregulation of these molecules, as reported in heterozygous knockouts (KO), does not affect the final signaling outcome. Glucose uptake in the muscle and adipose tissues via GLUT4, distal downstream of the insulin receptor-AKT pathway, is regulated by intracellular trafficking of GLUT4 to the plasma membrane by TBC1D4, and it gets impaired by mtROS via unknown mechanisms [16].

### 2.3. BCAA Metabolism Reduction in WAT as the Earliest Response to Obesity-Related Hyperinsulinemia and IR

Overnutrition leads to adipose tissue mass expansion either by hypertrophy (adipocyte size increase) or hyperplasia (increase in adipocyte number by preadipocyte differentiation and adipogenesis) [29]. Hyperplasia is considered healthy obesity, displaying well-organized growth with vascularization and producing beneficial adipokines such as leptin and adiponectin. In contrast, hypertrophy (i.e., pathologic obesity) causes fibrosis, inflammation, and necrosis, and is associated with ectopic lipid deposition. Generally, adipogenesis is considered to be protective against obesity-associated T2D. Subjects with a PPARγ variant that reduces its adipogenesis capacity have an increased risk of developing T2D [30], and PPARγ ligands are actually utilized as drugs for T2D.

**Figure 2:**
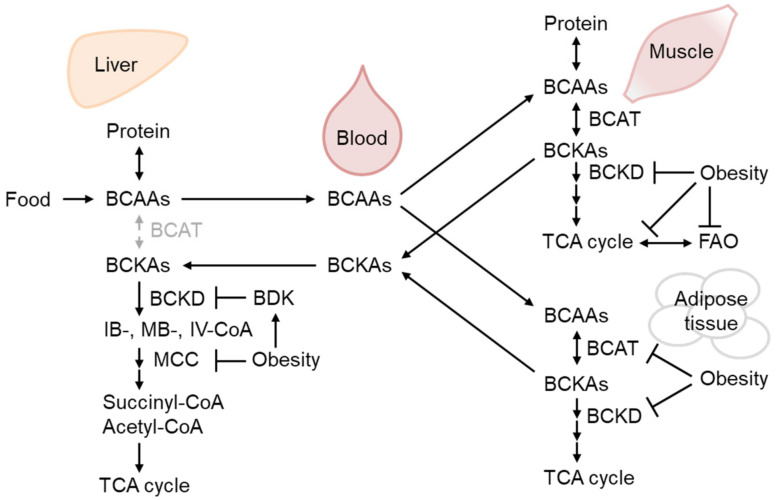
The regulation of branched-chain amino acid (BCAA) catabolism in obesity. BCAAs from dietary sources and protein breakdown are mainly oxidized by branched-chain aminotransferase (BCAT) in the muscle to respective ketoacids (BCKAs), which could be further oxidized by BCKA dehydrogenase (BCKD) and metabolized eventually into TCA cycle substrates, succinyl-CoA, and acetyl-CoA. Obesity decreases BCAA-catabolizing enzyme activity in the liver and skeletal muscle as well as their expression in the adipose tissue, leading to BCAA and BCKA accumulation in the blood. BCKD activity is inhibited by phosphorylation by BCKD kinase (BDK), which is increased in the liver of obese mice [47,48].

BCAAs are supplied by dietary ingredients, that are affected by gut microbiota metabolism, and by protein breakdown [31,32]. Importantly, adipogenesis utilizes a significant amount of BCAA as an energy source for preadipocyte differentiation [33], as do mature adipocytes [34]. After BCAAs are catabolized to corresponding ketoacids mainly in the muscle, branched-chain ketoacids are metabolized in the mitochondria by the branched-chain α-ketoacid dehydrogenase (BCKD) complex [35] (Figure 2). Zaganjor et al. demonstrate that Sirt4 regulates adipogenesis by regulating the activity of MCCC2, a subunit of methylcrotonyl-CoA carboxylase (MCC), which is a leucine metabolism rate-limiting enzyme downstream of BCKD [33]. Specific acyl-CoA species of leucine catabolism intermediates, such as 3-methylglutaryl-CoA and 3-hydroxy-3-methylglutaryl (HMG)-CoA, are highly reactive and acylate lysine residues of MCCC2. Importantly, Sirt4 is a specific lysine deacylase that catalyzes MCC deacylation and activates MCCC2 to enhance leucine catabolism [36]. Importantly, recent reports identified that the intermediates of leucine catabolism are essential for adipogenesis *in vivo* [37,38]. Sirt4 and enzymes of BCAA catabolism are downregulated in WAT during the early time course of ob/ob mice, which indicates that adipogenesis is downregulated by chronic hyper nutrition that might lead to unhealthy obesity [33]. A study of monozygotic twins described that BCAA catabolism-related gene expression is downregulated in the WAT of obese versus healthy participants, indicating that such downregulation involves, at least in part, transcriptional regulation [39].

The BCAA catabolism flux reportedly regulates fatty acid oxidation (FAO) in the skeletal muscle [40]. The study of inherited metabolic disease indicated that MCC deficiencies reportedly widely affect the transcriptome of mitochondrial energy metabolism, including those involved in electron transport and mitochondrial FAO [41]. Bioinformatic analysis in humans demonstrated that BCAA catabolism downregulation in the adipose tissue correlates with obesity-associated but not BMI-adjusted IR [42]. The authors subsequently demonstrated that BCAA catabolism enhancement alleviates IR. Moreover, the therapeutic effect of BCAA catabolism enhancement on IR was recently demonstrated in T2D patients [43]. Whether and how the downregulation of leucine catabolism affects the metabolism of fatty acids and contributes to IR should be clarified in the future.

Consistent with the above-mentioned reports, BCAA has emerged as the most reliable biomarker for obesity-associated IR and T2D [44], although a growing number of studies have demonstrated that BCAA supplementation positively affects metabolic health [45]. Pioneering metabolomic analysis demonstrated that BCAA or its metabolites are the most reliable markers of impaired fasting glucose after glucose in TwinsUK [44]. Plasma BCAA or their respective ketoacid metabolites are predictors of future T2D, with the latter being better markers. Interestingly, Guo et al. recently reported that low BCAA treatment in mice increases ceramide accumulation in the serum, indicating some connection between BCAA metabolism and ceramide accumulation [46].

Important reports in this section were cited in Table 1.

## 3. The Role of mtROS in IR

### 3.1. mtROS as the Cause of IR

**Figure 3:**
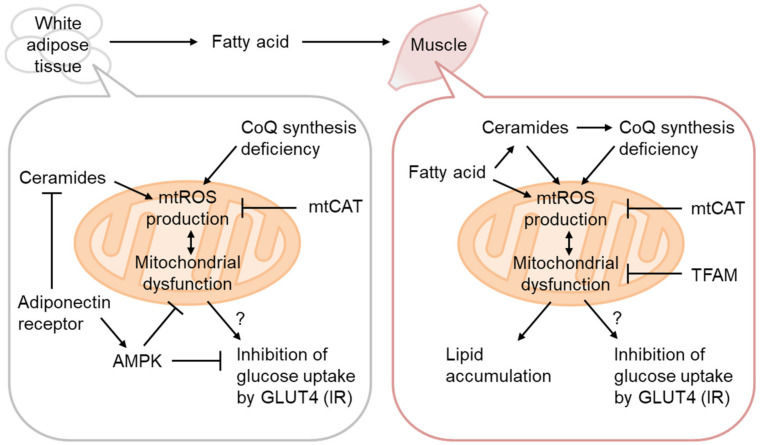
Mitochondrial ROS production in IR. Coenzyme Q (CoQ) synthetic pathway downregulation in IR increases H_2_O_2_ production from Complex II, rescued by CoQ supplementation or mitochondria-targeting catalase (mtCAT) expression. Adipocyte hypertrophy attenuates adiponectin signaling and the ceramidase activity of the adiponectin receptor, resulting in ceramide accumulation and mitochondrial dysfunction. mtROS production in the skeletal muscle is increased by excess fatty acid oxidation, either directly or indirectly, via ceramide accumulation and CoQ depletion. H_2_O_2_ production by mitochondria-targeting paraquat (MitoParaquat) can mimic muscle IR, reversed by mtCAT or TFAM expression. Mitochondrial dysfunction, rather than AKT inhibition by ceramide, affects GLUT4 translocation to the plasma membrane and inhibits glucose uptake.

Mitochondrial dysfunction, oxidative stress, and lipid accumulation are often associated with IR in the skeletal muscle [50]. The causative role of mitochondrial dysfunction has thus been mostly examined in this tissue [51] (Figure 3). As decreased OxPhos and lipid accumulation without enhancement of systemic lipolysis and signs of inflammation have been observed in the muscles of prediabetic lean IR people of the offspring of T2D patients [21], mitochondrial dysfunction might be causative for IR. It is known that a single-nucleotide polymorphism in arylamine *N*-acetyltransferase 2 (NAT2) causes IR in humans independent of BMI [52]. In *Nat* gene KO mice, mitochondrial dysfunction leads to ectopic lipid accumulation and mediates *NAT2* gene variant-mediated IR, which also supports mitochondrial dysfunction in IR etiology [53]. However, certain contracting results have also been published. For example, the high T2D prevalence in Asian Indians is associated with high OxPhos activity [54].

Given the adipose tissue-origin hypothesis of systemic IR during obesity-related T2D, IR in other tissues might be secondary to that in the adipose tissue. In the skeletal muscle of high-fat diet (HFD)-treated mice, mtROS production is heavily linked to IR [55]. Overexpression of mitochondria-targeting catalase (mtCAT) [55,56] or transcription factor A, mitochondria (TFAM) [57] reportedly prevents IR, supporting mitochondrial involvement in IR etiology. Lee et al. showed that HFD-induced IR, but not lipid-infusion-mediated IR, is reduced by mtCAT overexpression, indicating a non-direct effect of mtCAT on IR. He also showed increased oxygen consumption without an increase in ATP in mtCAT mice. Consistently, TFAM overexpression also increased FAT oxidation, accompanied by a higher β-oxidation capacity. Fazakerley et al. demonstrated that mtROS generation using mito-paraquat induces IR independently of altered oxidative phosphorylation [58]. In WAT, coenzyme Q synthetic enzyme downregulation is responsible for Complex II-related ROS generation [59]. Although the causal relationship between mtROS and IR has been extensively studied using animal models and *in vitro* cellular models [2], *in vivo* sources of mtROS and target characterization remain challenging, especially in humans.

### 3.2. Ceramides as mtROS Sources in Insulin Resistance

Ceramides are generated via *de novo* biosynthesis, sphingomyelin hydrolysis, the catabolism of complex sphingolipids, etc. [60]. It has been known that ceramide synthesis is activated by various stresses, including inflammation and oxidative stress, and that it regulates apoptosis, etc., but recent reports have placed its central role in the regulation of metabolism. Actually, several studies have indicated that inhibition of ceramide synthesis prevents diet-induced IR. Ceramides may act as gauges for surplus FAs, as serine and palmitoyl-CoA are the starting substrates of *de novo* ceramide synthesis. Various conditions reportedly provoke IR [50], most of which induce ceramide accumulation [60]. Therefore, ceramides are increasingly probable to mediate IR in various conditions. Since adiponectin receptors have ceramidase activity, downregulation of adiponectin in IR might also be associated with ceramide accumulation [61] (Figure 3). Ceramide accumulation reportedly increases mtROS via inhibition of respiratory complexes [62]. Consistently, ceramide biosynthesis downregulation improves IR in mice [63]. A recent study clarified that mitochondrial ceramide accumulation causes a decrease in mitochondrial coenzyme Q and subsequently compromises mitochondrial ETC activities, especially complex I [38]. However, the precise mechanisms of ceramide accumulation during obesity and IR require further investigation.

Important reports in this section were cited in Table 2.

## 4. Role of Nrf2 in Prevention of Obesity-Related IR and T2D

### 4.1. The Regulation of Oxidative Stress Response by Nrf2 

**Figure 4:**
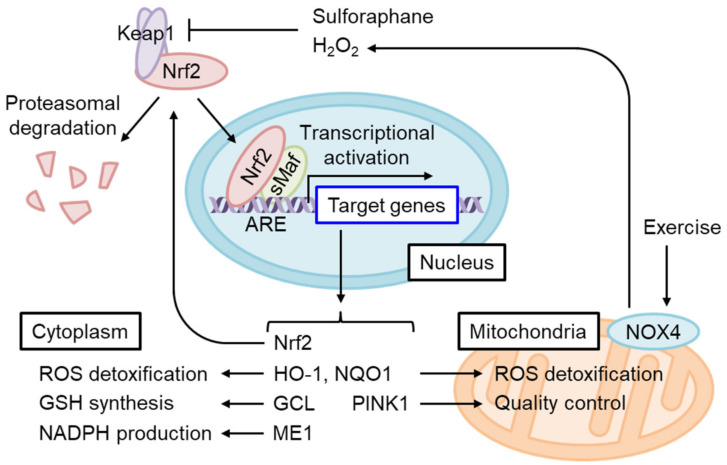
Nrf2 activation restores cytoplasmic and mitochondrial oxidative stress in IR. Nrf2 constitutively undergoes Keap1-mediated ubiquitination and proteasomal degradation under physiological redox states. Keap1 is inhibited by the adduction or oxidation of specific cysteine residues by electrophiles and by oxidative stress. Sulforaphane or H_2_O_2_ produced by exercise-stimulated NOX4 stabilizes and activates Nrf2. A heterodimer of Nrf2 and small Maf (sMaf) binds the antioxidant response element (ARE) and induces target gene transcription. ARE is present in the Nrf2 gene enhancer and increases Nrf2 mRNA levels in an auto-regulatory loop. Nrf2 target genes include those encoding enzymes that detoxify cytoplasmic ROS as well as electrophiles and produce antioxidants, while other genes regulate mitochondrial redox homeostasis and quality control.

**Figure 5:**
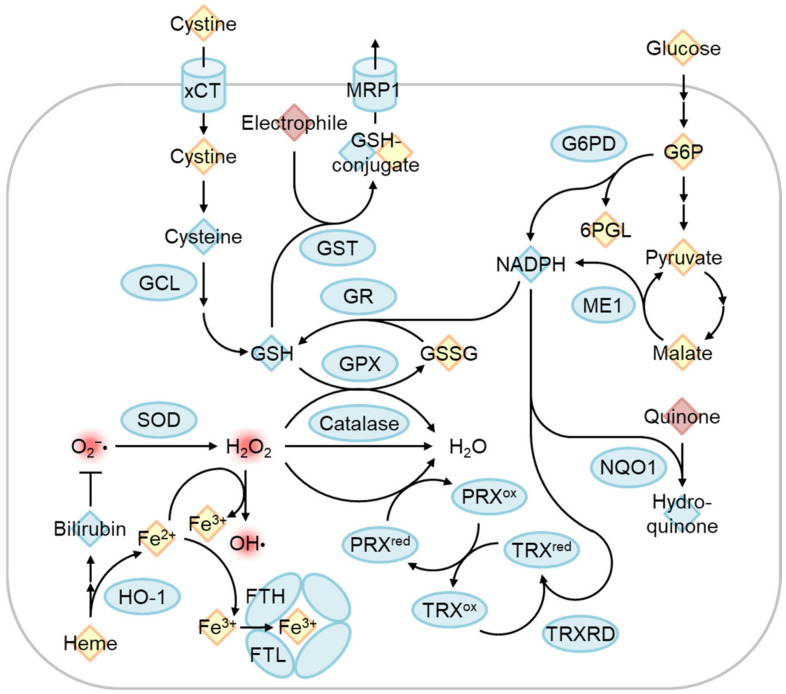
Regulation of oxidative stress response by Nrf2 target genes. The Nrf2-regulated gene product is indicated by the blue circle. Reactive oxygen species and electrophiles are indicated in red. Antioxidants and other chemicals were indicated as blue and yellow diamonds, respectively. Cystine/glutamate transporter (xCT) and glutamate-cysteine ligase (GCL) are involved in glutathione synthesis, and glutathione reductase recycles oxidized glutathione (GSSG) to reduced glutathione (GSH). GSH is used as an antioxidant, conjugation of electrophile by glutathione S-transferase (GST) to excrete via multidrug resistance-associated protein (MRP1), and reduction of H_2_O_2_ to H_2_O by glutathione peroxidase (GPX). H_2_O_2_ can be produced by superoxide (O_2_^−^•) dismutase (SOD) and is eliminated by the catalase and peroxiredoxin (PRX)/thioredoxin (TRX) systems. Free heme is degraded by heme oxygenase-1 (HO-1), and the degradation product bilirubin functions as an antioxidant. Released ferrous iron (Fe^2+^) can convert H_2_O_2_ into a hydroxyl radical (OH•) as known as the Fenton reaction. Fe^2+^ is oxidized to ferric iron (Fe^3+^) by the ferritin heavy chain (FTH) and stored inactively. Glucose-6-phosphate (G6P) dehydrogenase (G6PD) and malic enzyme 1 (ME1) produce NADPH, which is used for reactions by GR, TRX reductase (TRXRD), and NAD(P)H Quinone oxidoreductase 1 (NQO1). G6PD is a pentose phosphate pathway enzyme that produces 6-phosphogluconolactone (6PGL).

Nrf2 is a short-lived protein with a half-life of ~20 min under non-stressed conditions, submitted to Keap1-mediated ubiquitination and proteasomal degradation [7] (Figure 4). The oxidation of certain Keap1 cysteine residues by electrophiles or ROS induces Nrf2 accumulation and the dimerization of Nrf2 and small Maf (sMaf) in the nucleus. The Nrf2/sMaf heterodimer binds to the antioxidant response element (ARE) and induces the expression of various detoxification- and antioxidant synthesis-related genes (Figure 5). Nrf2 activates its own ARE-mediated transcription in the enhancer region to form a positive feedback loop [64]. Nrf2 contributes not only to the systemic detoxification of electrophiles and amelioration of oxidative stress in various tissues such as the liver and intestine but also to the exercise capacity of skeletal muscles [65], amelioration of inflammation, as well as adipocyte differentiation and metabolism [66], which are important for the etiology of IR. To discuss the role of Nrf2 in the prevention of IR and T2D, the main question should be whether Nrf2 activation has some impact on the above-mentioned pathways of obesity-related IR development.

### 4.2. The Role of Nrf2 Activation in WAT and Obesity

Several studies have examined how Nrf2 could affect obesity or IR using systemic *Nrf2* or *Keap1* knockdown (KD) mice (i.e., constitutive Nrf2 activation model). Both mice are reportedly resistant to HFD-induced obesity, with some contradicting results in the case of the former [67]. As Nrf2 in adipocytes and preadipocytes contributes to lipid metabolism, differentiation, and adipogenesis conducted by C/EBPβ and RXRα, Nrf2 is expectably involved in adipogenesis in response to HFD [66,68]. Although Nrf2 is generally required for HFD-induced obesity, as mentioned, constitutive Nrf2 activation by *Keap1* KD also inhibited HFD-induced obesity by decreasing PPARγ and C/EBPα [69]. As *Nrf2* KO affects HFD-induced obesity, the role of HFD-induced IR is impossible to analyze using this model without negating the effect of obesity. However, tissue-specific *Nrf2* KO in adipocytes, but not in hepatocytes, exacerbated HFD-induced IR [70]. Both of these mouse models exhibited comparable sensitivity to HFD-induced obesity, suggesting that Nrf2 displays a protective role against IR in adipose tissue. This result is partly consistent with the observation that Nrf2 is downregulated in the WAT of ob/ob mice, and both systemic and adipocyte-specific *Nrf2* deficiency exhibit more severe IR [71]. Therefore, the systemic *Nrf2* KO phenotype in pathological models should be carefully interpreted, and further research is needed to understand the tissue- and pathology-specific role of Nrf2. However, intriguingly, *Keap1* KO and various Nrf2 inducers display beneficial roles in HFD-induced obesity and IR (see below). Interestingly, Nrf2 inducer administration reduces WAT and restores reduced Nrf2 expression and its target genes both in the liver and adipose tissue [72,73].

### 4.3. The Role of Nrf2 in Pancreatic β-Cells

Pancreatic β-cells are known to be susceptible to oxidative stress due to the low-level expression of antioxidant enzymes and high ROS production coupled with glucose metabolism and insulin biosynthesis [74,75]. Although this review focuses on the early events of IR and the role of Nrf2 in pathophysiology [75], chronic hyperglycemia leads to oxidative stress and defective glucose-stimulated insulin secretion (GSIS) in β-cells (i.e., β-cell failure) during the progression to T2D following IR [76]. Baumel-Alterzon et al. showed that Nrf2 is activated by high glucose in *ex vivo* islet β-cells and also in mice fed HFD for 1 week [77]. β-cell-specific *Nrf2* KO mice fed HFD exhibited increased oxidative stress, decreased β-cell mass, and worsened glucose tolerance. In contrast, either β-cell-specific *Keap1* KO or administration of Nrf2 activator conferred resistance to β-cell dysfunction by HFD, implicating that the Nrf2 pathway contributes to the protection and therapeutic prevention of β-cells from oxidative stress [77].

### 4.4. The Role of Nrf2 in Mitochondrial Function

**Figure 6:**
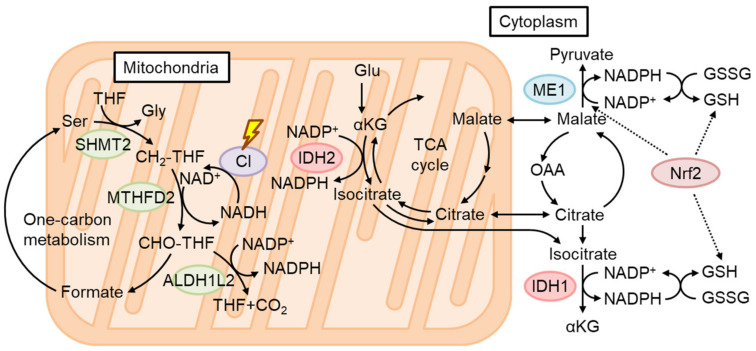
Mitochondrial NADPH production under restricted glucose and defective complex I. Under glucose restriction, decreased NADPH production via the pentose phosphate pathway induces ALDH1L2 expression, which produces NADPH, tetrahydrofolate (THF), and CO_2_ from 10-formyltetrahydrofolate (CHO-THF). Defects in respiratory complex I (CI) decrease NAD^+^ and thereby inhibit MTHFD2 of one-carbon metabolism. Defective CI also induces the reductive TCA cycle, and citrate and isocitrate exported to the cytoplasm can be used for NADPH production by IDH1. Overexpression of ME1 rescues cells with defective CI from oxidative stress and cell death. Nrf2 activation induces the expression of ME1 and GSH synthetic enzymes.

Nrf2 target genes mainly act in the protection of cytoplasmic oxidative stress, although certain target genes such as *HO-1*, *NQO1*, and *Pink1* might directly modify oxidative stress in the mitochondria [78] (Figure 4). On the other hand, excess mtROS, such as H_2_O_2_, might diffuse and cause oxidative stress in the cytoplasm. On this occasion, Nrf2 may protect against oxidative stress via the generation of GSH and upregulation of ROS detoxifying enzymes such as GPXs. However, excess ROS in some cases reduce Nrf2 translation, and therefore the Nrf2-mediated stress response is limited [79]. Interestingly, Balsa et al. screened the genes that rescue cell growth defects in human mitochondrial complex I mutant cells under restricted glucose availability [80]. Of note, they found that *ME1* overexpression most significantly rescues the phenotype. They found that the defect of complex I reduces one-carbon metabolism-mediated generation of mitochondrial NADPH production by reducing NAD^+^, which is a cofactor for MTHFD2 (Figure 6). Usually, NADPH production is thought to be compartmentalized in a subcellular compartment [81]. However, they showed that NADPH is shuttled to the cytoplasm via IDH1 and IDH2-related mechanisms and increases reduced GSH in the cytoplasm. Thus, the defect in complex I activity leads to a decrease in the GSH/GSSG ratio in the cytoplasm and provokes JNK-mediated apoptosis and inflammation. In this case, ME1-mediated generation of cytosolic NADPH greatly helps to rescue the oxidative stress observed in complex I deficiency. Considering that a decrease in mitochondrial coenzyme Q compromises mitochondrial ETC activities, especially complex I, during the generation of IR, Nrf2 activation should help to mitigate oxidative stress during IR, under which glucose availability is limited and the glucose-mediated pentose phosphate pathway is not available. Sulforaphane (SFN), isolated from cruciferous vegetables, is one of the most potent Nrf2 activators. Importantly, SFN inhibits ceramide synthetic enzymes such as serine palmitoyltransferase 3 in cultured hepatocytes and HFD-fed mice [82,83], although the dependency on Nrf2 was not examined.

### 4.5. Insulin Resistance Alleviation upon Nrf2 Activation

Nrf2 activation by Keap1 knockdown improves glucose intolerance by enhancing glycogen branching [84]. Indeed, SFN improves IR in HFD-fed mice [85,86]. Xirouchaki et al. demonstrated that NADPH oxidase 4 (NOX4) is induced by moderate or intense acute stress or exercise training in mice and humans that precedes *NOX2* induction when it occurs [87]. They demonstrated that skeletal muscle-specific *NOX4* decreases Nrf2, mitochondrial biogenesis-related genes, including PPARα, and mitochondrial biogenesis itself in in vitro cultured myotubes. Interestingly, SFN induces *NOX4* in an Nrf2-dependent manner, showing that SFN acts as an exercise mimic in the induction of the *NOX4* gene in the myotube. MtROS was enhanced in *NOX4*-deleted myotubes, and IR associated with obesity and old age was exacerbated in skeletal muscle-specific *NOX4* KO mice. Importantly, SFN-induced Nrf2 activation decreases mtROS-mediated oxidative stress and rescues decreases in insulin-induced AKT phosphorylation in *NOX4*-deficient myotubes. *GPX-1* deletion or mitochondrial antioxidant rescues IR in *NOX4*-deficient skeletal muscle. *GPX-1* deletion most probably increases H_2_O_2_ and activates Nrf2 signaling [87]. Thus, this study indicated that Nrf2 activation can improve mtROS-mediated IR. Mice fed HFD with glucoraphanin, a stable SFN precursor, displayed Nrf2-dependently improved obesity and IR by WAT browning and increased energy expenditure [86]. Therefore, intervention using Nrf2 inducers could potentially improve metabolic health in WAT and prevent IR and progression to T2D.

### 4.6. The Effect of Aging on T2D Etiology

Aging accelerates multiple age-related diseases, including T2D. Aging reportedly downregulates various cytoprotective factors, including the above-mentioned coenzyme Q [88] and Nrf2 [89]. Importantly, aging deteriorates mitochondrial function, as described in Harman’s free radical theory of aging. Among others, aging particularly deteriorates Complex I or IV activities. As Complex I mostly consists of nuclear- and mitochondrial-encoded subunits, reasonably efficient Complex I function is susceptible to aging. Moreover, Complex I catalyzes NADH oxidation to NAD^+^, which is important for anti-aging, acting as sirtuin co-factors. NAD^+^ reportedly declines with aging, and supplementation of its precursor, nicotinamide mononucleotide (NMN), improves T2D. The underlying mechanisms of mitochondrial dysfunction during aging might differ from tissue to tissue, although mitochondrial dysfunction is associated with a specific decline of Complex I activity in the heart [90]. Mitochondrial deterioration during aging may affect the sensitivity against IR, and an anti-aging strategy may be useful for the prevention of IR.

Important reports in this section were cited in Table 3.

## 5. Conclusions

**Figure 7:**
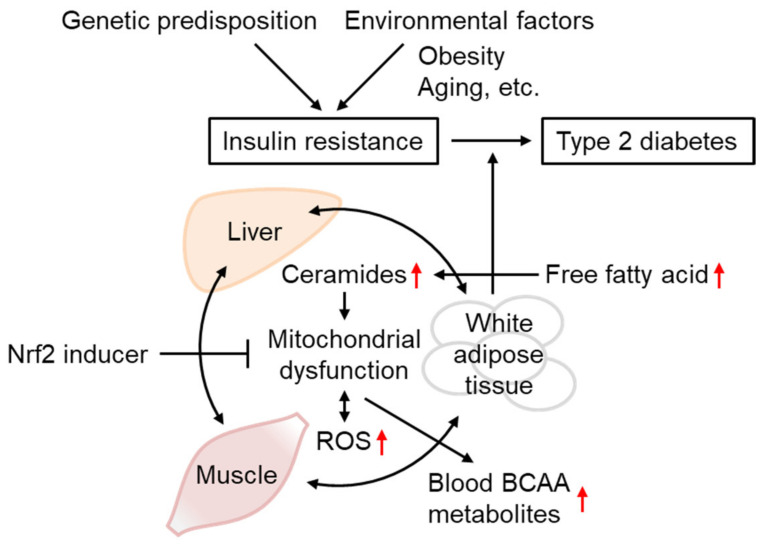
Insulin resistance progression and its prevention by Nrf2 inducers. The occurrence and progression of insulin resistance are affected both by genetic predisposition and environmental factors, including obesity and aging. IR is triggered by mitochondrial dysfunction and ROS overproduction, associated with ceramide accumulation. As IR in one tissue could propagate to other tissues, IR in the WAT and unhealthy obesity increase circulating free fatty acid levels, leading to depositions in the liver and muscles or conversion into ceramides, worsening the conditions that lead to type-2 diabetes. Defects in mitochondrial BCAA catabolism result in BCAA metabolite accumulation in the blood. Nrf2 inducer administration could improve IR through gene expression involved in antioxidant systems, mitochondrial function, and lipid metabolism.

Various results obtained in IR models support the importance of mtROS overproduction in IR and WAT metabolic abnormalities in disease progression toward other tissues and T2D (Figure 7). Although the functional role of Nrf2 in IR remains controversial based on studies of *Nrf2*-deficient animals, Nrf2 activation by SFN and other inducers is concordantly beneficial for IR in insulin-sensitive tissues, including WAT. As the accumulation of BCAA and its metabolites in the blood reflects mitochondrial metabolism in the muscles and WAT in the case of obesity, the administration of Nrf2 inducers might provide a potential preventive intervention strategy against IR and T2D. In order to better understand the etiology of human IR and its progression to T2D and determine the clinical efficacy of Nrf2 inducers, further cohort studies would be required to characterize the interaction of genetic and environmental factors, including obesity and aging (Figure 7). In addition, it is important to establish biomarkers or measurement methodologies for *in vivo* oxidative stress, mitochondrial dysfunction, and the state of Nrf2 activation. Finally, how mtROS regulates downstream molecules (e.g., GLUT4 trafficking) and whether Nrf2 can impact such actions of mtROS are not clear at present. Future experiments should clarify those questions.

## Figures and Tables

**Table 1:** Main reports associated with mitochondrial dysfunction, IR, and T2D pathogenesis.

Reports	References	Section
Hyperinsulinemia precedes IR	[17,18]	Section 2.1
Mitochondrial dysfunction and IR in lean T2D	[20,21]	Section 2.1
Genetic factors associated with T2D	[22,23]	Section 2.1
Selective IR in the liver	[25,26,27]	Section 2.2
Impairment of BCAA metabolism by obesity	[33,35,36,37,38,39,41,42,47,48,49]	Section 2.3
BCAA as a biomarker of obesity-associated IR	[44,45,46]	Section 2.3

**Table 2:** Main reports concerning mtROS associated with IR and T2D.

Reports	References	Section
Mitochondrial dysfunction in skeletal muscle is associated with IR	[21,50,51]	Section 3.1
NAT2 gene variant associated with IR etiology	[52,53]	Section 3.1
mtROS production is associated with IR etiology	[55,56,57,58,59]	Section 3.1
Ceramide accumulation leads to mtROS production	[38,46,50,60,61,62,63]	Section 2.3 and Section 3.2

**Table 3:** Main reports associated with Nrf2 and IR.

Reports	References	Section
Background of Nrf2 and the antioxidant system	[7,64,65,66]	Section 4.1
Systemic Nrf2 KO and obesity-induced IR	[66,67,68,69]	Section 4.2
Adipocyte-specific Nrf2 KO worsens obesity-induced IR	[70,71]	Section 4.2
Nrf2 activation improves obesity-induced IR	[72,73]	Section 4.2
Role of Nrf2 in pancreatic β-cell function	[77]	Section 4.3
Role of Nrf2 and its target genes in mitochondrial function	[78,81,82,83]	Section 4.4
IR alleviation by the Nrf2 activator	[84,86]	Section 4.5
NOX4-Nrf2 axis in muscle	[87]	Section 4.5
Aging and T2D etiology	[88,89,90]	Section 4.6

## Data Availability

Not applicable.
